# A Meta-Analysis on the Efficacy of Tai Chi in Patients with Parkinson's Disease between 2008 and 2014

**DOI:** 10.1155/2015/593263

**Published:** 2015-01-08

**Authors:** Ji Zhou, Tao Yin, Qian Gao, Xiao Cun Yang

**Affiliations:** ^1^Department of Tuina, Guang'anmen Hospital, China Academy of Chinese Medical Sciences, Beijing 100053, China; ^2^Department of Rehabilitation, Taian People's Hospital, Taian, Shandong 271000, China; ^3^Yueyang Hospital of Integrated Traditional Chinese and Western Medicine, Shanghai University of Traditional Chinese Medicine, Shanghai 201203, China

## Abstract

*Objective*. The purpose of this systematic review is to evaluate the evidence on the effect of Tai Chi for Parkinson's disease (PD).* Methods*. Six electronic databases up to June 2014 were searched. The methodological quality was assessed with PEDro scale. Standardised mean difference and 95% confidence intervals of random-effects model were calculated.* Results*. Nine studies were included in our review. The aggregated results are in favor of Tai Chi on improving motor function (*P* = 0.002) and balance (*P* < 0.00001) in patients with PD. However, there is no sufficient evidence to support or refute the value of Tai Chi on improving gait velocity (*P* = 0.11), stride length (*P* = 0.21), or quality of life (*P* = 0.40). And there is no valid evidence in follow-up effects of Tai Chi for PD. Conclusion. The current results suggest that Tai Chi can significantly improve the motor function and balance in patients with PD, but there is indeed not enough evidence to conclude that Tai Chi is effective for PD because of the small treatment effect, methodological flaws of eligible studies, and insufficient follow-up. Consequently, high-quality studies with long follow-up are warranted to confirm current beneficial findings.

## 1. Introduction

Parkinson's disease (PD) is a progressive neurodegenerative disease that is characterized by bradykinesia, resting tremor, rigidity, and postural instability [1]. And its prevalence is likely to increase among people aged over 50 years [2]. In China, it is estimated that the number of individuals with PD over the age of 50 was approximately 1.99 million in 2005 and it is expected to be nearly 4.94 million by the year 2030 [3]. The current management, especially for chronic and debilitating symptoms of PD, focuses on not only pharmacological therapy but also complementary and alternative medicine.

In the last decade, exercise interventions, as assisting pharmacological treatments of PD, showed desirable effects on improving balance, gait, and overall functional status of individuals with mild to moderate PD through experience-dependent neuroplasticity [4–6]. A large number of basic researches have also reported that exercises promoted cell proliferation and neuronal differentiation in animal models of PD [6–8]. Tai Chi, as a mind-body exercise consisting of proper breathing and slow controlled movements, has shown beneficial effects on improving muscle strength, balance, and motor function in older adults. It has been listed by the National Parkinson Foundation of the United States as one of the exercises to be beneficial for related clinical symptoms of PD [9]. However, the studies of Tai Chi for PD reported conflicting results. Li et al. reported significant improvements in balance, functional capacity, and falls after Tai Chi exercise [10]. In contrast, Amano et al. reported that Tai Chi was ineffective on either improving gait dysfunction or reducing Parkinsonian disability [11].

Therefore, the aim of this systematic review is to summarize and evaluate the evidence on the effectiveness of Tai Chi for PD. And the meta-analyses of Tai Chi for PD were conducted especially on motor function, balance, gait, and quality of life in individuals with PD.

## 2. Methods

### 2.1. Search Strategy

Relevant electronic databases were searched from the earliest date available to June 2014: PubMed, EMBASE, Cochrane Library, Wan Fang Data, Weipu Database for Chinese Technical Periodicals, and China Knowledge Resource Integrated Database (CNKI). The following search terms were used in various combinations: Parkinson's disease, Parkinson, Parkinsonism, Tai Chi, Taiji, and shadow boxing. And reference lists of relevant systematic reviews and primary studies were manually identified. In order to check for unpublished studies, trial registrations (WHO International Clinical Trials Registry Platform) and dissertations (Chinese Dissertation Full-Text Database and ProQuest Dissertations) were also searched. In addition, we have contacted the experts in the relevant field by e-mails.

### 2.2. Study Selection

The eligible studies must meet the following criteria: (1) study design: randomized controlled trials (RCTs); (2) the target population being diagnosed with PD; (3) types of interventions: Tai Chi compared with any comparator without Tai Chi relevant exercises; (4) the interesting outcomes including motor function, balance, gait, and quality of life; (5) the paper being available in either English or Chinese. The studies are excluded if (1) the primary intervention was Tai Chi plus other exercises; (2) the crossover studies did not report outcomes of the first phase so as to prevent any bias of carryover or order effects; (3) the studies are reported by only abstracts of conference proceedings without the detailed information.

After exclusion of duplicates, eligible studies were identified independently by two authors. Disagreements were resolved by a consensus after discussion. A third author was consulted in case of disagreements to improve accuracy.

### 2.3. Data Abstraction

For eligible studies, two reviewers independently extracted data based on a predesigned data extraction form including general information (first author, country, and year of publication, sample size, mean age, and Hoehn and Yahr stage of patients with PD), details of the interventions (treatment duration, style and dosage of Tai Chi, and comparison details), and main outcome assessments. The primary author was contacted by e-mails when relevant information was not reported. The authors resolved disagreements by discussions.

### 2.4. Methodological Quality Assessment

The methodological quality of eligible studies was assessed independently by two reviewers using PEDro scale. The PEDro scores ranged from 0 to 10 points, which has a fair-to-good reliability for RCTs of the physiotherapy in systematic reviews [19]. A higher score represents a better methodological quality. There is no disagreement between the reviewers regarding PEDro scores of eligible studies.

### 2.5. Statistical Analysis

Meta-analyses were carried out using Cochrane Collaboration software (Rev Man 5.1). In eligible studies, continuous data was combined using more conservative random-effects model for the expected heterogeneity. And standardized mean difference (SMD) and 95% confidence intervals (CI) were calculated in the meta-analyses. The *I* ^2^ statistic, a quantitative measure of inconsistency across studies, is employed in assessing heterogeneity. Heterogeneity is regarded high if the *I* ^2^ is greater than 75%. The studies with 2 similar control interventions had the groups combined with computational formula provided by the Cochrane handbook to create a single pairwise comparison. In addition, detailed subgroup analyses were conducted based on different outcome measures.

## 3. Results

### 3.1. Literature Search

**Figure 1:**
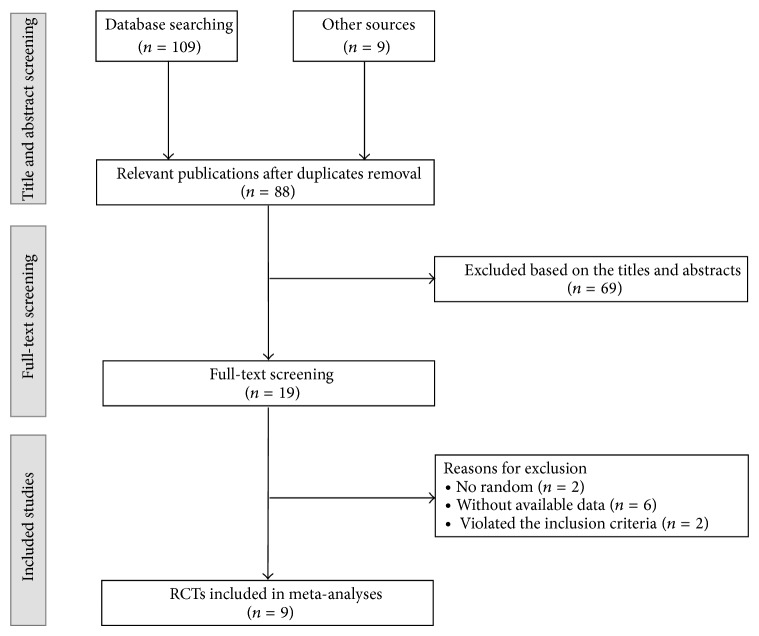
Flow chart for this meta-analysis. RCTs: randomized controlled trials.

A total of 118 relevant references were identified by our search strategy. After eliminating duplicate entries, the number of papers was reduced to 88. And 69 references were excluded by screening titles and abstracts. Finally, after assessing full-text papers of the remaining studies, 9 RCTs were included in our review [10–18]. Six studies were published in English [10–13, 16, 17] and three in Chinese [14, 15, 18]. The study selection process is depicted in Figure 1.

### 3.2. Study Characteristics

The principal characteristics of nine eligible studies are presented in Table 1. These studies were published between 2008 and 2014. The sample size ranged from 22 to 195 (total 569) with mean age of 66 years. And the Hoehn and Yahr stages ranged from 1 to 4. The common intervention in eligible trials is Yang-style Tai Chi. Compared interventions included no intervention [12, 16–18], walking [14, 15], dancing exercise [13], Qigong [11], and stretching/resistance training [10]. The treatment duration ranged from 4 to 24 weeks and exercise time of one session lasted 30–60 min.

### 3.3. Methodological Quality

Table 2 shows the PEDro scores of the eligible studies. The total scores range from 5 to 7 points. No studies employed concealed allocation, blinding of therapists, or blinding of participants that are difficult for the nonpharmacological clinical trials, but blinding of assessors is performed in all included studies [10–18]. The expulsion rate is higher than 15% in three studies [12–14]. Seven studies did not employ the intention-to-treat analysis due to cancelling the dropout data in the last results [12–18]. For the remaining criteria in PEDro scale, the included studies show a high methodological quality.

### 3.4. The Immediate Effects of Tai Chi for PD

#### 3.4.1. Motor Function

**Figure 2:**
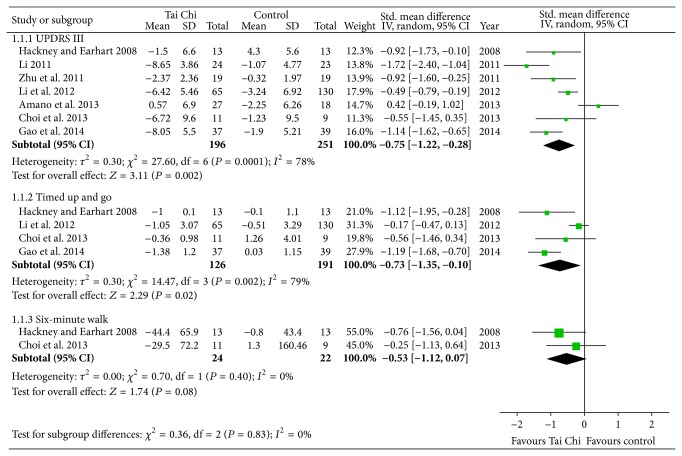
Forest plot showing the effect of Tai Chi on unified Parkinson's disease rating scale III (UPDRS III), timed up and go, and six-minute walk in patients with Parkinson's disease.

UPDRS III, clinician-scored monitored motor evaluation, is the most commonly used scale in the clinical study of PD. Seven studies assessed the effects of Tai Chi for PD on motor function by UPDRS III [10–12, 14–16, 18]. All of them were included in the meta-analysis. And the aggregated result has shown a significant benefit in favor of Tai Chi for PD on motor function (SMD, −0.75; 95% CI −1.22 to −0.28; *P* = 0.002; Figure 2).

The functional mobility in participants with PD was assessed using the timed up and go test (TUG) in four studies [10, 12, 16, 18]. The result of the meta-analysis showed that Tai Chi is associated with a statistical improving on TUG (SMD, −0.73; 95% CI −1.35 to −0.10; *P* = 0.02; Figure 2). However, the aggregate result of six-minute walk was not in favor of Tai Chi for PD (SMD, −0.53; 95% CI −1.12 to 0.07; *P* = 0.08; Figure 2) [12, 16].

#### 3.4.2. Balance

**Figure 3:**
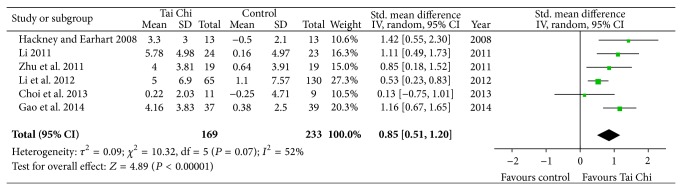
Forest plot showing the effect of Tai Chi on balance in patients with Parkinson's disease.

Six studies investigated the effect of Tai Chi on balance function in patients with PD [10, 12, 14–16, 18]. All studies showed favorable effects of Tai Chi on improving balance in individuals with PD. And the aggregated result was also in favor of Tai Chi on improving balance function (SMD, 0.85; 95% CI 0.51 to 1.20; *P* < 0.00001; Figure 3).

#### 3.4.3. Gait

**Figure 4:**
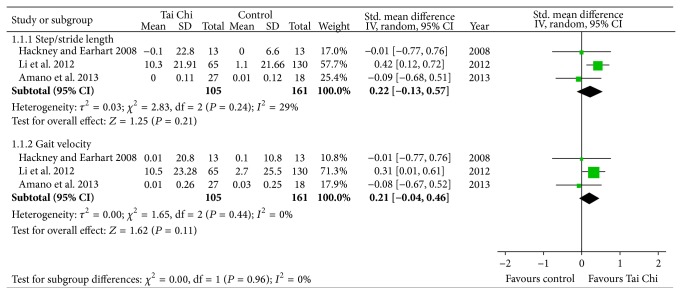
Forest plot showing the effect of Tai Chi on gait in patients with Parkinson's disease.

Three studies assessed the effect of Tai Chi on gait function in individuals with PD [10–12]. Two of them reported that there are no differences on gait between Tai Chi and control interventions [11, 12]. And the results of the meta-analysis also showed that Tai Chi is not associated with significant improvements on stride length (SMD, 0.22; 95% CI −0.13 to 0.57; *P* = 0.21; Figure 4) [10–12] or gait velocity (SMD, 0.21; 95% CI −0.04 to 0.46; *P* = 0.11; Figure 4) [10–12].

#### 3.4.4. Quality of Life

**Figure 5:**
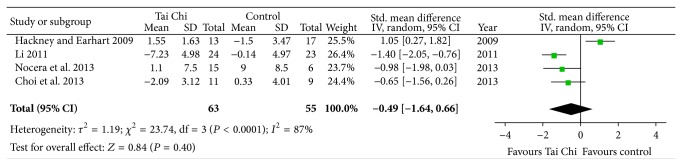
Forest plot showing the effect of Tai Chi on quality of life in patients with Parkinson's disease.

Four studies investigated the effectiveness of Tai Chi on quality of life in individuals with PD [13, 14, 16, 17]. Although two of them reported that Tai Chi group has significant better scores than control groups on Parkinson's disease questionnaire-39 [14, 17], the aggregate result showed that Tai Chi is not associated with a statistical improving on quality of life in individuals with PD (SMD, −0.49; 95% CI −1.64 to 0.66; *P* = 0.40; Figure 5) [13, 14, 16, 17].

### 3.5. The Follow-Up Effects of Tai Chi for PD

Only two studies evaluated the follow-up effects of Tai Chi for PD [10, 18]. One study reported that the main gains are maintained at the 3-month postintervention follow-up [10]. And the participants in Tai Chi group have fewer falls than those in the stretching group and in the resistance training group. The other one also reported that the incidence of falls among patients in Tai Chi group significantly decreases compared with control group (21.6% versus 48.7%) at the 6-month follow-up [18].

## 4. Discussion

The main purpose of this systematic review is to evaluate the evidence of Tai Chi for PD. The primary finding is that there is beneficial evidence of Tai Chi on improving motor function and balance in patients with PD. However, there is no evidence that Tai Chi is more effective on improving gait and quality of life in individuals with PD. And there is not enough evidence on follow-up effects of Tai Chi for PD.

This is a comprehensive systematic review that has evaluated the current evidence in the effect of Tai Chi for PD. In our systematic review, the detailed meta-analyses of Tai Chi for PD were performed on motor function, balance, gait, and quality of life. Hence our systematic review has shown the objective evidence that Tai Chi had better immediate effects on improving motor function and balance in patients with PD. And current reviews are consistent with our findings [20, 21]. What is more, the strict inclusion criteria increased the confidence in our results. Only RCTs, with the intention of minimizing risk of bias, were eligible in our review. Although other study designs can also contribute useful information to the evidence of Tai Chi for PD, more risks of bias may affect aggregated results in the meta-analyses. What is more, only studies with detailed and validated information in outcome measures were included in our review for conducting valuable meta-analyses.

Our main findings are different from the previous reviews [22, 23]. Lee and colleagues conclude that the evidence is insufficient to suggest Tai Chi is an effective modality for PD [23]. In their qualitative review, there were 3 RCTs [24–26], 1 nonrandomised controlled trial [27], and 3 uncontrolled clinical trials [28–30] published from 1997 to 2007. Furthermore, 3 RCTs were published only as abstracts which had not been formally reviewed due to lacking essential details. One suspected reason for this difference is that only RCTs with detailed data were eligible in our systematic review. And a large number of full-texts of RCTs were published from 2008 to 2014 [10–18]. Hence these new studies were included in our systematic review and formally peer-reviewed. Another possible explanation for the difference is that our systematic review is the first systematic review with a pooled estimate of Tai Chi for PD. Hence our systematic review shows the objective evidence of Tai Chi for PD because any strictly qualitative review may be more subjective than the meta-analyses. Overall, detailed meta-analyses on the effect of Tai Chi for PD have strengthened the confidence in our systematic review.

Tai Chi is beneficial for PD, especially for balance and motor function. This may be related with the improvements on coordination, cognitive function, stepping strategy, and flexibility. In Pei et al.'s study, Tai Chi practitioners showed better eye-hand coordination and movement control [31]. Indeed, during Tai Chi exercise, substantial postural control, mental concentration, and diaphragmatic breathing are emphasized [32, 33]. So Tai Chi usually is considered as a coordination exercise resulting in better postural and balance control. Moreover, emerging evidence indicates a relationship between coordination of movements and better cognitive function [34]. Tai Chi, as a mind-body exercise, offers positive cognitive benefits, such as executive function, memory, and learning [35, 36]. In addition, previous studies indicate that Tai Chi training significantly enhances balance by more effective mechanisms controlling stepping strategies [37, 38]. Tai Chi can increase flexibility, muscle strength, and endurance [39, 40]. Balance and motor function may depend on muscle strength and flexibility of lower and upper extremities.

### 4.1. Limitation

There are some potential limitations in our review. First, there is the degree of uncertainty in locating relevant studies because of limited retrieving resources, language barrier, and publication bias. Second, there are a small number of eligible studies in current review. It should be noted that there are methodological flaws in these trials. The therapists and patients were not blinded. Moreover, 5 trials used no intervention as a comparator. Although most studies employed blinding assessors to make up for these deficiencies, the bias cannot be avoided due to the placebo effect, which may affect the stability of current results.

In addition, there are statistically significant outcomes in the meta-analyses, but these results should be explained carefully. Tai Chi shows better improvements on motor function than control therapies, but the size of treatment effect is small, especially for UPDRS III. In some meta-analyses, the power is also small due to the small number of eligible studies. The six-minute walk and gait were analyzed based on two trials and three ones, respectively. Moreover, the synthetic results may also be affected by styles (Yang-style, Sun-style, etc.) and parameters (duration, frequency, dosage, etc.) of Tai Chi in our meta-analyses. The current results must be strongly challenged because of insufficient follow-up.

## 5. Conclusion

Although Tai Chi shows statistically significant improvements on motor function and balance for patients with PD in the current meta-analyses, there is indeed not enough evidence to conclude that Tai Chi is effective for PD because of the small treatment effect, methodological flaws of the eligible studies, and insufficient follow-up. Consequently, large-scale, high-quality RCTs with long follow-up are warranted to confirm current findings of Tai Chi for PD.

## Figures and Tables

**Table 1:** Characteristics of randomized controlled trials of Tai Chi for Parkinson's disease.

First author, year, country	Sample size	Mean age (year)	Hoehn and Yahr stage	Treatment duration (week)	Main outcome assessments	Experimental group intervention	Control group intervention
Hackney, 2008, US [12]	33	64	1.5–3	10–13	UPDRS III, BBS, gait, TUG, six-minute walk	Yang-style Tai Chi (60 min/20 sessions)	No intervention
Hackney, 2009, US [13]	75	67	1–3	13	PDQ-39	Yang-style Tai Chi (60 min/20 sessions)	(1) Tango (2) Waltz/Foxtrot (3) No intervention (60 min/20 sessions)
Li, 2011, China [14]	56	68	2.5–3	8	UPDRS III, BBS, PDQ-39	Tai Chi (30–45 min/80 sessions)	Walking (40 min/80 sessions)
Zhu, 2011, China [15]	40	64	1-2	4	UPDRS III, BBS	Tai Chi (30–45 min/40 sessions)	Walking (40 min/40 sessions)
Li, 2012, US [10]	195	69	1–4	24	UPDRS III, gait, TUG, functional-reach test	Tai Chi (60 min/48 sessions)	(1) Stretching (2) Resistance training (60 min/48 sessions)
Amano, 2013, US [11]	45	66	2-3	16	UPDRS III, gait	Yang-style Tai Chi (60 min/32–48 sessions)	(1) Qigong (60 min/32 sessions) (2) No intervention
Choi, 2013, US [16]	22	63	1-2	12	UPDRS, TUG, gait, six-minute walk, one-leg standing	Tai Chi (60 min/36 sessions)	No intervention
Nocera, 2013, US [17]	23	66	2-3	16	Cognitive-executive function, PDQ-39	Yang-style Tai Chi (60 min/48 sessions)	No intervention
Gao, 2014, China [18]	80	69	1–4	12	UPDRS III, BBS, TUG	Yang-style Tai Chi (60 min/36 sessions)	No intervention

UPDRS: unified Parkinson's disease rating scale; BBS: berg balance scale; TUG: timed up and go; PDQ-39: Parkinson's disease questionnaire-39.

**Table 2:** PEDro scales of included randomized controlled trials.

Study	Eligibility criteria	Random allocation	Concealed allocation	Similar at baseline	Subjects blinded	Therapists blinded	Assessors blinded	<15% dropouts	Intention-to-treat analysis	Between-group comparisons	Point measures and variability data	Total
Hackney and Earhart 2008 [12]	1	1	0	1	0	0	1	0	0	1	1	5
Hackney and Earhart 2009 [13]	1	1	0	1	0	0	1	0	0	1	1	5
Li 2011 [14]	1	1	0	1	0	0	1	0	0	1	1	6
Zhu et al. 2011 [15]	1	1	0	1	0	0	1	1	0	1	1	6
Li et al. 2012 [10]	1	1	0	1	0	0	1	1	1	1	1	7
Amano et al. 2013 [11]	1	1	0	1	0	0	1	1	1	1	1	7
Choi et al. 2013 [16]	1	1	0	1	0	0	1	1	0	1	1	6
Nocera et al. 2013 [17]	1	1	0	1	0	0	1	1	0	1	1	6
Gao et al. 2014 [18]	1	1	0	1	0	0	1	1	0	1	1	6

0: does not meet the criteria; 1: meets the criteria. Criteria (without eligibility criteria) were used to calculate the total PEDro score.
